# Considerations to forgo systemic treatment in patients with advanced esophageal or gastric cancer: A real‐world evidence study

**DOI:** 10.1002/ijc.35314

**Published:** 2025-01-09

**Authors:** Ellis Slotman, Marieke Pape, Hanneke W. M. van Laarhoven, Roos E. Pouw, Yvette M. van der Linden, Rob H. A. Verhoeven, Sabine Siesling, Heidi P. Fransen, Natasja J. H. Raijmakers

**Affiliations:** ^1^ Department of Research and Development Netherlands Comprehensive Cancer Organisation Utrecht The Netherlands; ^2^ Department of Health Technology and Services Research University of Twente, Technical Medical Centre Enschede The Netherlands; ^3^ Medical Oncology Amsterdam UMC location University of Amsterdam Amsterdam The Netherlands; ^4^ Cancer Treatment and Quality of Life Cancer Center Amsterdam Amsterdam The Netherlands; ^5^ Department of Gastroenterology and Hepatology Amsterdam University Medical Centers, location VUmc Amsterdam The Netherlands; ^6^ Centre of Expertise in Palliative Care Leiden University Medical Centre Leiden The Netherlands; ^7^ Department of Radiotherapy Leiden University Medical Centre Leiden The Netherlands

**Keywords:** advanced, best supportive care, esophageal cancer, gastric cancer, systemic treatment

## Abstract

The majority of patients with advanced esophageal or gastric cancer do not start palliative systemic treatment. To gain insight into the considerations underlying the decision not to start systemic treatment, we analyzed characteristics of patients starting and not starting systemic treatment, reasons for not starting systemic treatment, and receipt of local palliative treatments on a nationwide scale. Patients diagnosed with advanced esophageal or gastric cancer between 2015 and 2021 were included (*n* = 10,948). Survival was compared using propensity score matching on patient and disease characteristics. Most patients did not start systemic treatment (esophageal cancer 59%; gastric cancer 64%). These patients were generally older, more often female, had more comorbidities and a worse performance status. The main reason for not starting systemic treatment was patient or family preference (35%). Among patients who did not start systemic treatment, 47% (esophageal) and 19% (gastric), received local palliative treatment, most commonly radiotherapy. Patients who did not start systemic treatment had worse median overall survival compared to patients who did start (esophageal cancer 2.9 months vs. 8.9 months; gastric cancer 2.2 vs. 8.2 months). These findings indicate that patient condition and disease burden are important aspects in systemic treatment decisions. However, patient or family preference was the main reason for not starting systemic treatment, highlighting that their priorities also strongly influence the decision. Systemic treatment did show to be associated with improved overall survival in matched patients, and therefore adequately weighing treatment risks and benefits based on real world data against patient preferences is of utmost importance.

## Graphical abstract

What's new?

Most patients with advanced esophageal or gastric cancer do not start palliative systemic treatment. The considerations and rationale in forgoing systemic treatment remain unclear. This nationwide study of patients diagnosed with advanced esophageal or gastric cancer between 2015 and 2021 indicates that the main reason for not starting palliative systemic treatment is patient preference. Overall survival was significantly longer with systemic treatment in matched patients with similar prognostic profiles at diagnosis. The findings highlight the importance of balancing treatment benefits with patient priorities based on real‐world data, and underscore the need for personalized decision‐making in advanced esophageal and gastric cancer.

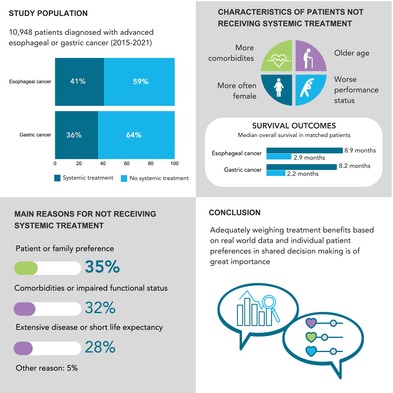

## 1 INTRODUCTION

Esophageal and gastric cancer are both among the top 10 most common cancers worldwide, with over 600,000 and 1 million new cases diagnosed each year, respectively.^1^ The prognosis is poor, especially when patients are diagnosed at an advanced stage, with a median survival time of 4 months for patients with metastatic gastric cancer and 5 months for patients with metastatic esophageal cancer.^2^ Curative options are usually not viable and the preferred approach is to initiate palliative systemic treatment to hopefully prolong life and also manage symptoms.^3^, ^4^ If the main presenting symptom is dysphagia, then palliative radiotherapy is the preferred local treatment, and initiation of systemic treatment may follow.

However, more than half of the patients with advanced esophageal or gastric cancer do not start any palliative systemic treatment.^5^, ^6^ Since most studies focus only on those patients undergoing systemic treatment, little research has been devoted to the considerations and rationale for not receiving systemic treatment. Patient and disease characteristics and the expected survival benefit are likely important determinants of the decision to start or forgo systemic treatment. However, survival evidence is often derived from clinical trials that enroll relatively fit patients, and it may be difficult to generalize this evidence to all patients seen in daily clinical practice. Studies in patients with advanced pancreatic and ovarian cancer showed that patient preference was the main reason for not receiving cancer‐directed treatment.^7^, ^8^ Other factors such as personal beliefs and values and socioeconomic factors are also known to play a role in treatment decisions.^9^ In addition, the provision of local palliative treatments plays an important role in the management of patients with advanced esophageal or gastric cancer,^3^, ^4^ and may be an appropriate alternative to systemic treatment depending on the symptoms and treatment goals. Therefore, another consideration in not starting systemic treatment may be the decision to treat symptoms only locally.

To better understand the treatment decisions and considerations in patients with advanced esophageal or gastric cancer who do not start systemic treatment, this population‐based study aimed to describe the characteristics of patients who did and did not start systemic treatment and to gain insight into the reasons for not receiving systemic treatment. In addition, this study aimed to assess the use of local palliative therapies in patients not receiving systemic treatment and the characteristics associated with their use and to compare survival in patients starting and not starting systemic treatment.

## 2 METHODS

### 2.1 Study population and data

**FIGURE 1:**
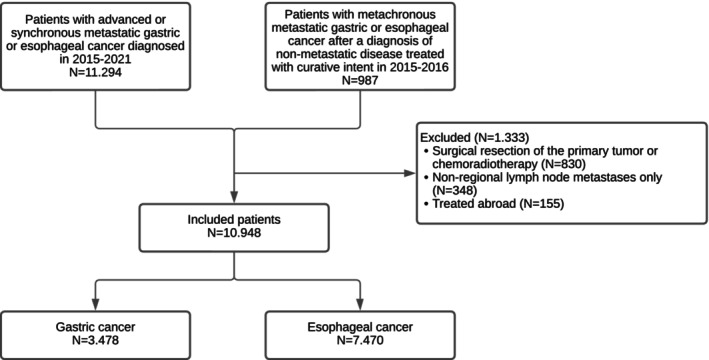
Flowchart of the patient selection.

Patients diagnosed with unresectable advanced (cT_4b_cN_all_cM_0_) or synchronous metastatic (cT_all_cN_all_cM_1_) disease in 2015–2021 and patients diagnosed with metachronous metastatic disease after primary diagnosis and curative treatment of non‐metastatic disease (cT_1‐4a_, _X_cN_all_cM_0_) in 2015–2016 were selected from the Netherlands Cancer Registry (NCR) (Figure 1).^10^ The NCR is based on notification of all newly diagnosed malignancies in the Netherlands by the national automated pathology archive. Additional information on diagnosis, stage, and treatment is extracted from medical records by data managers. Data on metachronous metastatic disease and subsequent treatment were collected retrospectively by data managers directly from the patient files in the second half of 2019. For all included patients, data on patient, tumor, and hospital characteristics, initial treatments (systemic treatment and non‐systemic local treatments), reasons for not starting systemic treatment, and survival were obtained from the NCR.

Synchronous metastatic disease was defined as a diagnosis of metastases prior to or within 5 days of initiation of systemic treatment to account for potential delays in pathologic confirmation of metastases. If patients did not receive systemic treatment, synchronous disease was defined as a diagnosis of metastases within 6 weeks of the primary diagnosis. Metachronous metastatic disease was defined as a diagnosis of metastases after the end of treatment with curative intent (i.e., resection or definitive chemoradiotherapy) for non‐metastatic disease. To account for delays in pathologic confirmation, metastases had to be diagnosed at least 5 days after the resection.

Patients who received surgical resection of the primary tumor or chemoradiotherapy (*N* = 830) or patients with non‐regional lymph node metastases limited to the head and neck region (*N* = 348) were excluded to avoid including patients with a potentially curative treatment intent (Figure 1).^11^ Patients treated outside the Netherlands (*N* = 155) were also excluded.

### 2.2 Hospital characteristics

Hospital volume was divided into quartiles based on the number of patients diagnosed with unresectable advanced or synchronous metastatic esophageal or gastric cancer per hospital in 2015–2020 (Q1 ≤ 68 patients, Q2 69–107 patients, Q3 108–169 patients, Q4 ≥ 170 patients). Surgical treatment of esophageal or gastric cancer is centralized in 20 hospitals in the Netherlands (i.e., center of expertise), which also perform multidisciplinary consultations involving various specialists to collaboratively discuss all treatment options. In this study, the hospital of first contact and diagnosis was classified as a center of expertise if it was one of these 20 hospitals.

### 2.3 Treatment characteristics

Type of therapy was classified as systemic treatment or no systemic treatment. For patients not receiving systemic treatment, data on local palliative treatments was collected, categorized as: radiotherapy on the primary tumor, radiotherapy on metastases, gastric bypass, or stent placement (categories not mutually exclusive). The main reason for not starting systemic treatment was recorded in the NCR following four predefined options in descending order of priority: (1) comorbidities or impaired performance status; (2) extensive disease or short life expectancy; (3) patient or family preference; (4) other reason. Data on vital status were obtained by an annual linkage of the NCR to the Dutch Personal Records Database and were available until February 1, 2022.

### 2.4 Statistical analyses

All analyses were stratified by primary tumor site: esophageal cancer or gastric cancer. Characteristics of patients starting and not starting systemic treatment were compared using chi‐squared tests. Reasons for not starting systemic treatment and the proportion of patients receiving local palliative treatments were presented using descriptive statistics. The reason for not starting systemic treatment was additionally stratified by performance status and type of disease (unresectable advanced/synchronous metastatic or metachronous metastatic). Logistic regression analyses were performed to identify independent factors associated with receipt of any local palliative treatment. These analyses were initially performed for patients with synchronous metastatic disease since they comprised over 80% of the study population. To identify differences between synchronous and metachronous metastatic disease, the regression analysis was additionally performed in patients with metachronous metastatic esophageal cancer, but not gastric cancer due to small numbers. To further investigate the factors influencing clinical decision‐making with respect to the type of local palliative treatment for dysphagia, regression analyses were performed separately for stent placement and local radiotherapy in patients with synchronous metastatic esophageal cancer.

Overall survival (OS) was assessed from primary diagnosis (cT4b and synchronous metastatic disease) or date of first metastasis (metachronous metastatic disease) until death or end of follow‐up. OS was compared between the systemic and no systemic treatment groups using Kaplan–Meier survival curves and Cox proportional hazards analyses. To account for prognostic differences between patients in the systemic and no systemic treatment group, nearest neighbor (1:1) propensity score‐matching was used to match patients in both groups based on the following 12 patient and disease characteristics at diagnosis: age, sex, hemoglobin levels, lactate dehydrogenase levels, weight loss (patient's usual weight minus weight at diagnosis in kilograms), disease type (cT4b, synchronous metastatic or metachronous metastatic disease), performance status, number of comorbidities, number and localization of metastases, Lauren classification.^12^ and histology. Patients with unknown values on these variables were excluded from the matching procedure. The caliper was set to 0.01 of the standard deviation of the logit of the propensity score to minimize within‐pair differences. Standardized mean difference was used to examine the balance of the variables between groups after matching. A sensitivity analysis was performed excluding patients who died within 30 days of diagnosis to account for an expected timely death, logically depriving the patient of the chance to start with systemic treatment. All analyses were performed using Stata version 17.0 software (StataCorp LLC, College Station, Texas, USA). A two‐sided *p*‐value <.05 was considered statistically significant.

## 3 RESULTS

### 3.1 Characteristics of patients not receiving systemic treatment

A total of 7470 patients with advanced esophageal cancer and 3478 patients with advanced gastric cancer were included (Figure 1). In the majority of patients, no systemic treatment was started (esophageal cancer: 4414 (59%); gastric cancer: 2216 (64%)). The most prominent differences in patient characteristics between patients who did and did not start systemic treatment for both esophageal and gastric cancer were that patients not starting systemic treatment were older, had more comorbidities, and worse performance status (Table 1). Furthermore, patients with unresectable advanced or metachronous metastatic disease did start systemic treatment less often compared to patients with synchronous metastatic disease in both esophageal and gastric cancer. For esophageal cancer, patients who did not start systemic treatment more often had squamous cell carcinoma compared to patients who did not start systemic treatment (19% vs. 9%, *p* < .001). Patients who did not start systemic treatment were also slightly more often diagnosed in a center of expertise (esophageal cancer: 34% vs. 30%, *p* < .001; gastric cancer 33% vs. 30%, *p* = .009).

**TABLE 1:** Characteristics of patients with advanced gastric and esophageal cancer diagnosed in 2015–2021 who did and did not start systemic treatment.

	Esophageal cancer			Gastric cancer		
	Systemic treatment	No systemic treatment		Systemic treatment	No systemic treatment	
	*N* (%)	*N* (%)	*P*‐value	*N* (%)	*N* (%)	*P*‐value
Number of patients	3056	4414		1262	2216	
Patient characteristics at diagnosis						
Age; median (IQR)	64 (59–71)	72 (65–79)	<.001	66 (56–73)	76 (68–82)	<.001
Hemoglobin (mmol/L); median (IQR)	8 (8–9)	8 (7–9)	<.001	8 (7–9)	7 (6–8)	<.001
LDH (U/L); median (IQR)	217 (176–309)	227 (179–372)	<.001	200 (168–251)	226 (178–331)	<.001
Weight loss (kg); median (IQR)	6 (2–10)	7 (4–12)	<.001	7 (3–10)	7 (4–10)	.64
Sex						
Male	2485 (81)	3303 (75)	<.001	768 (61)	1304 (59)	.25
Female	571 (19)	1111 (25)		494 (39)	912 (41)	
Number of comorbidities						
0	1685 (55)	1782 (40)	<.001	720 (57)	899 (41)	<.001
1	906 (30)	1462 (33)		368 (29)	694 (31)	
≥2	373 (12)	1003 (23)		144 (11)	529 (24)	
Unknown	92 (3)	167 (4)		30 (2)	94 (4)	
WHO performance status						
0–1	2178 (71)	1368 (31)	<.001	832 (66)	548 (25)	<.001
≥2	309 (10)	1250 (28)		173 (14)	563 (25)	
Unknown	569 (19)	1796 (41)		257 (20)	1105 (50)	
Disease characteristics at diagnosis						
Disease type						
Unresectable advanced	29 (1)	195 (4)	<.001	30 (2)	102 (5)	<.001
Synchronous metastatic	2790 (91)	3725 (84)		1185 (94)	1941 (88)	
Metachronous metastatic	237 (8)	494 (11)		47 (4)	173 (8)	
Histology						
Adenocarcinoma	2629 (86)	3409 (77)	<.001	1224 (97)	2161 (98)	.35
Squamous cell carcinoma	268 (9)	826 (19)		NA	NA	
Other	159 (5)	179 (4)		38 (3)	55 (2)	
Tumor differentiation						
Well/moderate	932 (31)	1132 (26)	<.001	175 (14)	303 (14)	.18
Poorly/undifferentiated	1117 (37)	1597 (36)		517 (41)	844 (38)	
Unknown	1007 (33)	1685 (38)		570 (45)	1069 (48)	
Lauren classification						
Intestinal	1125 (43)	1144 (33)	<.001	333 (27)	535 (25)	<.001
Diffuse	359 (14)	506 (15)		635 (52)	901 (42)	
Mixed	66 (3)	43 (1)		38 (3)	56 (3)	
Indeterminate	73 (3)	112 (3)		18 (1)	37 (2)	
Unknown	1006 (38)	1604 (47)		200 (16)	632 (29)	
Not applicable (histology other than adenocarcinoma)	427	1005		38	55	
Number of distant metastatic sites						
0–1	1772 (58)	2654 (60)	.003	871 (69)	1487 (67)	.05
2	877 (29)	1114 (25)		295 (23)	505 (23)	
≥3	407 (13)	646 (15)		96 (8)	224 (10)	
Localization of metastases (not mutually exclusive)						
Extra regional lymph node metastases	1459 (47)	1891 (43)	<.001	382 (30)	673 (30)	.95
Liver metastases	1745 (57)	2059 (47)	<.001	375 (30)	810 (37)	<.001
Peritoneal metastases	392 (13)	563 (13)	.92	748 (59)	1144 (52)	<.001
Lung metastases	695 (23)	1204 (27)	<.001	96 (8)	242 (11)	.002
Bone metastases	567 (19)	937 (21)	.005	109 (9)	186 (8)	.80
Other sites	484 (16)	967 (22)	<.001	104 (8)	250 (11)	.004
Characteristics of the hospital of diagnosis						
Center of expertise						
Yes	2151 (70)	2909 (66)	<.001	883 (70)	1488 (67)	.08
Diagnostic hospital volume						
Q1 (≤68)	266 (9)	345 (8)	.36	93 (7)	152 (7)	.13
Q2 (69–107)	621 (20)	869 (20)		266 (21)	400 (18)	
Q3 (108–169)	877 (29)	1264 (29)		356 (28)	643 (29)	
Q4 (≥170)	1292 (42)	1936 (43)		547 (43)	1021 (46)	

### 3.2 Main reason for not starting systemic treatment

**FIGURE 2:**
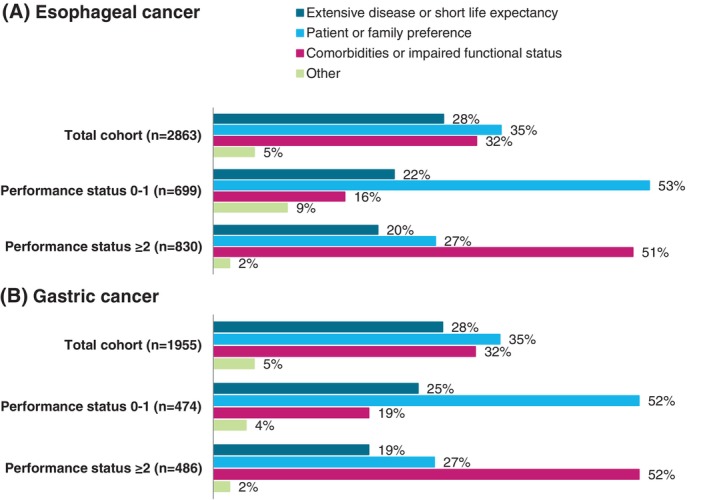
Main reason for not receiving systemic treatment in patients with advanced esophageal (A) or gastric (B) cancer for all patients and stratified by performance status. Data are shown only for patients for whom the reason was recorded.

The main reason for not starting systemic treatment was reported in 4.818 of the 6.630 patients (72%) who did not start systemic treatment. For both esophageal (*n* = 2863) and gastric (*n* = 1955) cancer, the most frequently reported reason was patient or family preference (35%), followed by comorbidities or impaired functional status (32%) and extensive disease or short life expectancy (28%) (Figure 2). In patients with a good performance status (0–1), the most common reason for not starting systemic treatment was patient or family preference (esophageal cancer 54%; gastric cancer 52%), whereas in patients with a poor performance status (≥2) the impaired functional status was the most commonly reported reason (esophageal cancer 50%; gastric cancer 52%). In patients with primary advanced or synchronous metastatic disease, the most commonly reported reason for not receiving treatment was patient or family preference (35%), whereas the most commonly reported reason in patients with metachronous metastatic disease was comorbidities or impaired functional status (esophageal cancer 34%; gastric cancer 33%) (Figure S1).

### 3.3 Local palliative treatments

Among patients with esophageal cancer who did not start systemic treatment, 47% received local palliative treatment. This was 19% for gastric cancer. The most frequently received treatment was radiotherapy on the primary tumor (esophageal cancer 31%; gastric cancer 8%), followed by stent placement (esophageal cancer 13%; gastric cancer 5%) and radiotherapy on metastases (esophageal cancer 10%; gastric cancer 2%). Bypass surgery was performed in 5% of patients with advanced gastric cancer.

In patients with synchronous metastatic esophageal cancer, a worse performance status, peritoneal metastases, or liver metastases were independently associated with lower odds of receiving local palliative treatment, whereas increasing age and a squamous cell carcinoma were associated with higher odds of receiving local palliative treatment (Table 2). When stratified according to stent placement and local radiotherapy, worse performance status and presence of liver metastases remained associated with lower odds of local radiotherapy but not with stent placement (Table S1). Increased weight loss was associated with lower odds of local radiotherapy, but higher odds of stent placement. For both patients with esophageal and gastric cancer, the presence of bone metastases was associated with higher odds of receiving local palliative treatment (Table 2). Patients for whom weight loss or performance status were unknown had lower odds of receiving local palliative treatment. In metachronous metastatic esophageal cancer, worse performance status, peritoneal metastases, or liver metastases were independently associated with lower odds of receiving local palliative treatment (Table S2).

**TABLE 2:** Logistic regression analyses for the probability of receiving local palliative treatment in patients with synchronous esophageal and gastric cancer who did not start systemic treatment.

	Esophageal cancer				Gastric cancer			
	Univariable		Multivariable		Univariable		Multivariable	
	OR (95%CI)	*P*‐value	OR (95%CI)	*P*‐value	OR (95%CI)	*P*‐value	OR (95%CI)	*P*‐value
Patient characteristics at diagnosis								
Age	1.00 (1.00–1.01)	.03	1.01 (1.00–1.01)	.002	1.00 (0.99–1.01)	.49	1.00 (0.99–1.02)	.11
Sex								
Male	Ref		Ref		Ref		Ref	
Female	0.92 (0.80–1.07)	.33	0.91 (0.78–1.08)	.31	0.82 (0.64–1.04)	.11	0.79 (0.61–1.01)	.06
Weight loss^a^								
<= 10%	Ref		Ref		Ref		Ref	
>10%	0.89 (0.74–1.07)	.24	0.96 (0.74–1.17)	.73	0.73 (0.53–1.02)	.07	0.79 (0.56–1.10)	.17
Unknown	0.66 (0.56–0.76)	<.001	0.80 (0.68–0.95)	.01	0.53 (0.40–0.69)	<.001	0.60 (0.45–0.80)	<.001
Number of comorbidities								
0	Ref		Ref		Ref		Ref	
1	1.13 (0.97–1.32)	.09	1.08 (0.92–1.28)	.31	0.93 (0.70–1.22)	.61	0.86 (0.65–1.15)	.31
≥2	1.07 (0.90–1.26)	.40	1.01 (0.84–1.21)	.91	0.84 (0.62–1.14)	.27	0.76 (0.55–1.05)	.10
Unknown	0.82 (0.58–1.16)	.27	1.00 (0.68–1.46)	.97	0.95 (0.54–1.69)	.88	1.06 (0.59–1.91)	.82
WHO performance status								
0–1	Ref		Ref		Ref		Ref	
≥2	0.53 (0.44–0.61)	<.001	0.53 (0.44–0.63)	<.001	1.02 (0.75–1.37)	.89	1.07 (0.78–1.46)	.63
Unknown	0.31 (0.27–0.37)	<.001	0.32 (0.35–0.38)	<.001	0.53 (0.40–0.71)	<.001	0.59 (0.44–0.80)	.001
Disease characteristics at diagnosis								
Histology								
Adenocarcinoma	Ref		Ref		Ref		Ref	
Squamous cell carcinoma	1.76 (1.47–2.11)	<.001	1.52 (1.25–1.85)	<.001	NA	NA	NA	NA
Other	0.65 (0.47–0.90)	.01	0.70 (0.49–0.99)	.05	0.83 (0.39–1.80)	0.65	0.82 (0.37–1.79)	.62
Localization of metastases								
Extraregional lymph node metastases	1.20 (1.05–1.37)	.004	1.01 (0.88–1.17)	.79	0.98 (0.76–1.26)	.907	0.88 (0.67–1.15)	.35
Liver metastases	0.48 (0.42–0.55)	<.001	0.49 (0.45–0.56)	<.001	0.93 (0.73–1.18)	.58	0.85 (0.65–1.12)	.27
Peritoneal metastases	0.42 (0.24–0.52)	<0.001	0.40 (0.32–0.50)	<.001	0.88 (0.69–1.10)	.27	0.76 (0.58–1.01)	.06
Lung metastases	1.05 (0.91–1.22)	.42	1.03 (0.88–1.20)	.67	0.86 (0.59–1.26)	.46	0.88 90.59–1.29)	.52
Bone metastases	1.44 (1.23–1.69)	<.001	1.43 (1.20–1.69)	<.001	1.47 (1.01–2.13)	.04	1.51 (1.02–2.26)	.04
Other sites	0.78 (0.66–0.92)	.003	0.76 (0.64–0.91)	.003	0.51 (0.33–0.80)	.003	0.53 (0.33–0.83)	.006
Characteristics of the hospital of diagnosis								
Center of expertise								
Yes	1.02 (0.88–1.17)	.75	0.97 (0.83–1.14)	.78	1.25 (0.98–1.60)	.07	1.19 (0.91–1.54)	.19
Hospital volume								
Q1 (≤68)	Ref		Ref		Ref		Ref	
Q2 (69–107)	0.88 (0.66–1.17)	.38	0.87 (0.64–1.18)	.34	1.13 (0.67–1.89)	.62	1.10 (0.65–1.87)	.69
Q3 (108–169)	0.80 (0.61–1.05)	.11	0.80 (0.60–1.07)	.14	0.92 (0.56–1.50)	.74	0.88 (0.53–1.46)	.63
Q4 (≥170)	0.78 (0.60–1.01)	.07	0.69 (0.52–0.93)	.01	1.00 (0.62–1.61)	.98	0.86 (0.52–1.40)	.54

^a^ <=10% meaning that patients' weight at diagnosis was 10% or less below their usual weight. >10% meaning that the patient's weight at diagnosis was more than 10% below their usual weight.

### 3.4 Survival

**FIGURE 3:**
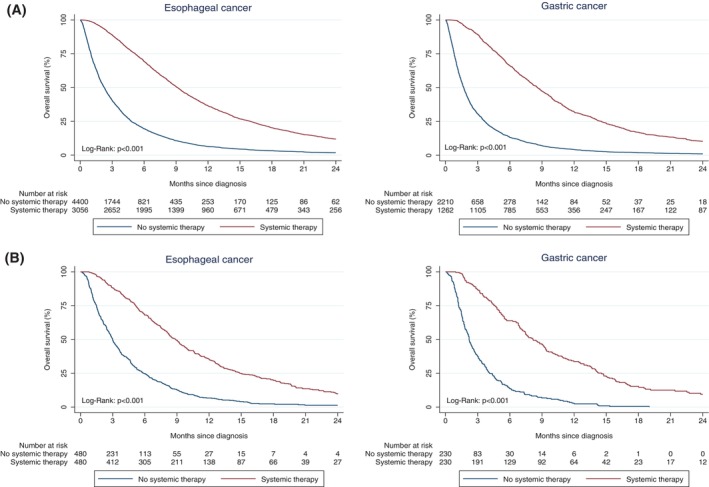
Overall survival in patients with advanced esophageal or gastric cancer who did and did not start systemic treatment in (A) the overall cohort and (B) a matched cohort. Patients were matched on age, sex, hemoglobin and lactate dehydrogenase levels, weight loss (patient's usual weight minus weight at diagnosis in kilograms), disease type (cT4b, synchronous metastatic or metachronous metastatic disease), performance status, number of comorbidities, number and localization of metastases, Lauren classification and histology.

In advanced esophageal cancer, median OS was 2.3 months in patients who did not start systemic treatment versus 9.1 months in patients who did start systemic treatment (HR 3.03, (95% confidence interval [CI] 2.88–3.19), *p* < .001) (Table 3/Figure 3A). For gastric cancer, this was 1.7 months versus 8.6 months respectively (HR 3.51 [95%CI 3.25–3.79], *p* < .001). After matching 480 patients with esophageal cancer and 230 patients with gastric cancer who started systemic treatment with the same number of patients who did not start systemic treatment based on patient and disease characteristics (Table S3), median OS remained lower for patients who did not start systemic treatment (esophageal cancer 3.0 vs. 8.8 months, HR 2.91 [95%CI 2.53–3.35], *p* < .001; esophageal cancer 2.2 months vs. 8.1 months, HR 3.95 [95%CI 3.19–4.89], *p* < .001) (Table 3/Figure 3B). Similar results were observed after excluding patients who died within 30 days of diagnosis (Figure S2).

**TABLE 3:** Cox regression for overall survival in patients with advanced esophageal or gastric cancer who did and did not start systemic treatment in the overall cohort and a matched cohort with similar prognostic profiles at diagnosis.

	Unmatched cohort				Matched cohort^a^			
	*N*	Median OS	Univariable regression		*N*	Median OS	Univariable regression	
			HR (95% CI)	*P*‐value			HR (95% CI)	*P*‐value
Esophageal cancer								
Systemic therapy	4414	9.3	Ref		480	9.2	Ref	
No Systemic therapy	3056	2.6	2.81 (2.63–2.98)	<.001	480	2.9	3.19 (2.66–3.84)	<.001
Gastric cancer								
Systemic therapy	1262	8.8	Ref		230	8.5	Ref	
No Systemic therapy	2216	1.7	3.54 (3.33–3.75)	<.001	230	2.5	3.52 (3.00–4.13)	<.001

^a^ Patients were matched on age, sex, hemoglobin and lactate dehydrogenase levels, weight loss (patient's usual weight minus weight at diagnosis in kilograms), disease type (cT4b, synchronous metastatic or metachronous metastatic disease), performance status, number of comorbidities, number and localization of metastases, Lauren classification and histology.

## 4 DISCUSSION

This population‐based study shows that patients with advanced esophageal or gastric cancer who do not start palliative systemic treatment differ significantly from those who do start palliative systemic treatment in how and where they present at diagnosis and in their overall survival. The main reasons for not starting palliative systemic treatment were the patient or family preference or poor functional status. In the absence of palliative systemic treatment, about one‐third of patients received other local palliative treatment, the most common of which was radiotherapy. Poor performance status and the presence of liver metastases were associated with lower odds of receiving local palliative treatment.

It was expected that the patient's physical condition would be an important determinant in the decision not to start systemic treatment. This is confirmed in this study, as patients who did not start systemic treatment were older, had more comorbidities, and worse performance status. Worse functional status was also the second most commonly reported reason for not starting systemic treatment, and in patients with poor performance status specifically, it was the most commonly reported reason, further highlighting that the patient's condition is an important determinant in forgoing systemic treatment. In addition, patients with esophageal cancer who did not start systemic treatment more often had squamous cell carcinoma, which may be related to the fact that evidence for palliative systemic therapy in esophageal squamous cell carcinoma has been limited.^13^, ^14^, ^15^, ^16^ Furthermore, patients who were first seen and diagnosed in a center of expertise were more likely to not start systemic treatment. One possible explanation may be that physicians who specialize in the treatment of esophageal or gastric cancer have become more adept at selecting patients who are fit and likely to benefit most from systemic treatment and may therefore be more likely to deviate from guideline‐recommended treatment. In addition, physicians may be more likely to opt for less invasive local palliative treatments as a result of multidisciplinary consultation.

Although physical fitness was shown to be an important consideration in the decision to start systemic treatment, it is noteworthy that one‐quarter to one‐third of patients who did not start systemic treatment had a good performance status. For these patients, the main reason they did not start systemic treatment was because of their own preferences. This suggests that patients are involved in the shared decision‐making process and highlights that for patients there is more to the decision of starting a systemic treatment than whether they will be able to tolerate it based on their physical condition. Previous studies have shown that treatment decisions are also influenced by factors such as the level of social support, personal or family experience with treatment, beliefs in non‐conventional treatments, and the value placed on quality of life and comfort.^17^, ^18^ In addition, cultural and religious aspects can play an important role in palliative treatment decisions by influencing trust in the healthcare system, patient and clinician attitudes toward death, and attitudes toward shared decision‐making and advance care planning.^19^, ^20^, ^21^ For example, patients from a Western background and culture may be more likely to forgo palliative systemic treatments because of a greater emphasis on supportive care, and more open communication about end‐of‐life issues, whereas in other cultures, such as some Eastern cultures, patients may feel a stronger incentive to start treatment, influenced by family and societal expectations and different attitudes to illness and death.^21^, ^22^, ^23^ However, even among Western countries, the proportion of patients receiving systemic treatment varies widely,^24^ suggesting that the differences between countries are not solely related to culture or religion. The results of this study underscore the importance of shared decision‐making regarding the initiation of systemic treatment in patients with advanced esophageal or gastric cancer, taking into account the patient's wishes, values, and needs.

Expected quality of life and survival benefits are considered important determinants in systemic treatment decisions. Several studies using cancer registry data have shown better survival in patients with advanced esophageal or gastric cancer who receive systemic treatment.^5^, ^6^, ^25^ This study showed that palliative systemic treatment was associated with a modest improvement in overall survival, also after matching patients with similar prognostic profiles at diagnosis, thereby eliminating some of the confounding by indication present in the previous studies. These results suggest that in daily clinical practice, patients benefit from systemic treatment in terms of prolonging life. However, there are other considerations, such as quality of life and maintaining independence, that can be important goals for patients.^26^ Systemic treatment is known to be associated with burden to the patient and family, including treatment‐related toxicity.^27^ However, previous studies do suggest that health‐related quality of life generally remains relatively stable during systemic treatment for advanced esophageal or gastric cancer.^28^, ^29^, ^30^ Systemic treatment can also be associated with time toxicity for patients and relatives. Patients spend part of the last phase of their lives undergoing treatment and traveling in and out of the hospital, and relatives often need to accompany patients on these visits. Therefore, it is important to discuss with each patient whether the expected survival benefit outweighs the potential burden on the patient and relatives. As part of this discussion, personalized predictions of treatment and survival outcomes can be of added value in helping to make informed decisions.^31^, ^32^, ^33^, ^34^

In the absence of palliative systemic treatment, 20%–50% of the patients with advanced esophageal or gastric cancer received one or more local palliative treatments. Several patient and disease characteristics seem to be associated with receiving local palliative treatment. In patients with esophageal cancer, poorer performance status and the presence of peritoneal or liver metastases were associated with a lower likelihood of receiving local palliative treatment, primarily a lower likelihood of receiving local radiotherapy. In addition, greater weight loss at diagnosis decreased the odds of receiving local radiotherapy, whereas it increased the odds of receiving stent placement. This is likely due to the fact that stent placement provides more rapid relief of dysphagia, whereas radiotherapy provides slower but more durable relief, leading to the recommendation of local radiotherapy for all patients with a relatively good prognosis and stent placement for patients with a poorer prognosis and short life expectancy.^35^ Patients with esophageal squamous cell carcinoma were also more likely to receive local palliative treatment compared to patients with esophageal adenocarcinoma, which may be related to the fact that squamous cell carcinomas tend to be more sensitive to radiotherapy.^36^ Patients with esophageal or gastric cancer and bone metastases were more likely to receive local palliative treatments, as expected since painful bone metastases are an important indication for palliative radiotherapy. This study found no clear associations between the receipt of local palliative treatments and hospital characteristics, suggesting that patients have an equal chance of receiving these treatments regardless of where they present at diagnosis.

## 5 STRENGTHS AND LIMITATIONS

The main strength of this study is the use of nationwide population‐based data, thus providing a representative reflection of unselected patients seen in clinical practice. However, some limitations should be noted. First, information on symptom burden is not available in the NCR. This makes it impossible to determine how many patients had an indication for local palliative treatment and to adjust for symptom burden in the regression analyses. Second, the NCR does not include information on symptom management with medication, nor on nutritional support and specialist palliative care, which are also important aspects of supportive care for patients with advanced esophagogastric esophageal or gastric cancer. Third, only one reason for not receiving treatment is recorded in the NCR. Although the item is recorded by trained and experienced administrators following strict guidelines, this assessment may still be subject to interpretation because often a combination of reasons plays a role in the decision‐making process and reasons may be interrelated. Lastly, data were incomplete for some variables, such as weight loss and performance status, which may have resulted in suboptimal adjustment in the multivariable regression models. More accurate reporting of these variables in electronic health records, regardless of whether a patient receives a certain treatment, would help to better assess real‐world treatment patterns and outcomes for all patients with advanced esophageal and gastric cancer seen in daily clinical practice, thereby providing better insights for informed shared decision making.

## 6 CONCLUSION

This population‐based study showed that the vast majority of patients with advanced esophageal or gastric cancer do not start any systemic treatment. Patient, tumor, and hospital characteristics are important factors in the decision to initiate palliative systemic treatment, as they differ significantly between patients who did and did not initiate systemic treatment. However, this study also showed that patient preference was the main reason for not starting systemic treatment, suggesting that patients are involved in the decision‐making process and highlighting that the decision not to start systemic treatment appears to depend not only on clinical parameters but also to a large extent on the priorities of patients and their families. OS was significantly longer with systemic treatment in patients with similar prognostic profiles at diagnosis, demonstrating the potential of systemic treatment to prolong life in the real‐world patient population. Taken together, these findings underscore the importance of weighing the risks and benefits of treatment based on real‐world data against patient preferences in making appropriate treatment decisions.

## AUTHOR CONTRIBUTIONS

**Ellis Slotman:** Conceptualization; writing – original draft; formal analysis; data curation; visualization; methodology. **Marieke Pape:** Conceptualization; methodology; writing – review and editing. **Hanneke W. M. van Laarhoven:** Writing – review and editing. **Roos E. Pouw:** Writing – review and editing. **Yvette M. van der Linden:** Writing – review and editing; supervision. **Rob H. A. Verhoeven:** Conceptualization; writing – review and editing. **Sabine Siesling:** Writing – review and editing; supervision. **Heidi P. Fransen:** Conceptualization; writing – review and editing; supervision. **Natasja J. H. Raijmakers:** Conceptualization; writing – review and editing; supervision.

## CONFLICT OF INTEREST STATEMENT

HWM van Laarhoven reports research funding and/or medication supply from Amphera, Anocca, Astellas, AstraZeneca, Beigene, Boehringer, Daiichy‐Sankyo, Dragonfly, MSD, Myeloid, ORCA, and Servier, served a consultant or advisory role for Auristone, Incyte, Merck, Myeloid, Servier, and reports a speaker role for Astellas, Beigene, Benecke, BMS, Daiichy‐Sankyo, JAAP, Medtalks, Novartis, Springer, Travel Congress Management B.V. RHA Verhoeven has received research grants from Bristol Myers Squibb and Amgen and performed consultancy for Daiichi Sankyo, all paid to the institute. The other authors report no conflicts of interest related to this study.

## ETHICS STATEMENT

The study was approved by the Privacy Review Board of the NCR (reference number K22.245).

## Supporting information


**Data S1.** Supporting Information.

## Data Availability

The data that were used for this study are available upon reasonable request from the Netherlands Cancer Registry. Requests can be made through the application form which can be found at: https://iknl.nl/en/ncr/apply-for-data. Further information is available from the corresponding author upon request.
