# Supplementary material for: Considerations to forgo systemic treatment in patients with advanced esophageal or gastric cancer: A real‐world evidence study
Source: Int J Cancer. 2025 Jan 9;156(10):1950–60. doi: 10.1002/ijc.35314 (PMC11924308; doi:10.1002/ijc.35314)
Supplement: Supplementary file 1 — Data S1. Supporting Information. [file IJC-156-1950-s001.pdf]

Considerations to forgo systemic treatment in patients with advanced esophageal or gastric cancer: a real-world evidence study

Ellis Slotman, Marieke Pape, Hanneke WM van Laarhoven, Roos vE Pouw, Yvette M van der Linden, Rob HA Verhoeven, Sabine Siesling, Heidi P Fransen, Natasja JH Raijmakers

Table of contents

Page 2/3 - Table S1: Logistic regression analyses for the probability of receiving stent placement or local radiotherapy in patients with synchronous metastatic esophageal cancer who received no systemic treatment.

Page 4 - Table S2: Logistic regression analyses for the probability of receiving symptom-directed treatment in patients with metachronous metastatic esophageal cancer who received no systemic treatment.

Page 5/6 - Table S3: Characteristics of patients with advanced gastric and esophageal cancer diagnosed in 2015-2021 who received and who did not receive systemic treatment (matched cohort).

Page 7 – Figure S1: Main reason for forgoing treatment in patients with advanced esophageal (a) or gastric (b) cancer stratified by type of disease (primary advanced/synchronous metastatic or metachronous metastatic. Data are shown only for patients for whom the reason was recorded.

Page 8 - Figure S2: Kaplan Meijer curves of overall survival in patients with advanced esophagogastric cancer who received systemic treatment and who did not receive systemic treatment in a matched cohort with similar prognostic profiles at diagnosis, excluding patients who died within one month of diagnosis.

Table S1: Logistic regression analyses for the probability of receiving stent placement or local radiotherapy in patients with synchronous metastatic esophageal cancer who received no systemic treatment.

	**Stent placement**				**Local radiotherapy**			
	**Univariable**		**Multivariable**		**Univariable**		**Multivariable**	
	**OR (95%CI)**	**P-value**	**OR (95%CI)**	**P-value**	**OR (95%CI)**	**P-value**	**OR (95%CI)**	**P-value**
**Patient characteristics at diagnosis**								
Age	1.01 (1.00-1.02)	0.01	1.01 (1.00-1.02)	0.01	1.00 (0.99-1.01)	0.10	1.00 (1.00-1.01)	0.03
Sex								
Male	Ref		Ref		Ref		Ref	
Female	1.06 (0.85-1.31)	0.57	0.97 (0.78-1.22)	0.83	0.91 (0.78-1.06)	0.26	0.90 (0.76-1.08)	0.28
Weight loss*								
<= 10%	Ref		Ref		Ref		Ref	
>10%	1.37 (1.06-1.79)	0.02	1.35 (1.03-1.77)	0.03	0.76 (0.63-0.92)	0.005	0.84 (0.69-1.03)	0.11
Unknown	1.12 (0.89-1.42)	0.29	1.12 (0.88-1.42)	0.35	0.55 (0.47-0.65)	<0.001	0.70 (0.59-0.84)	<0.001
Number of comorbidities								
0	Ref		Ref		Ref		Ref	
1	1.05 (0.84-1.31)	0.61	0.99 (0.79-1.24)	0.98	1.12 (0.96-1.32)	0.14	1.07 (0.90-1.27)	0.42
≥2	1.01 (0.79-1.30)	0.88	0.91 (0.70-1.18)	0.43	1.14 (0.96-1.36)	0.13	1.08 (0.89-1.32)	0.41
Unknown	0.66 (0.36-1.20)	0.18	0.68 (0.37-1.25)	0.21	0.85 (0.58-1.24)	0.41	1.13 (0.75-1.71)	0.55
WHO performance status								
0-1	Ref		Ref		Ref		Ref	
≥2	1.12 (0.87-1.43)	0.35	1.08 (0.84-1.40)	0.51	1.02 (0.75-1.37)	0.89	1.07 (0.78-1.46)	0.63
Unknown	1.18 (0.94-1.48)	0.15	1.19 (0.94-1.50)	0.14	0.53 (0.40-0.71)	<0.001	0.59 (0.44-0.80)	0.001
**Disease characteristics at diagnosis**								
Histology								
Adenocarcinoma	Ref		Ref		Ref		Ref	
Squamous cell carcinoma	1.49 (1.17-1.88)	0.001	1.40 (1.09-1.80)	0.007	1.64 (1.38-1.96)*	<0.001	1.43 (1.17-1.74)*	<0.001
Other	1.08 (0.68-1.72)	0.72	1.02 (0.64-1.63)	0.92	0.56 (0.38-0.82)*	0.003	0.60 (0.40-0.89)*	0.01

Localization of metastases								
Extraregional lymph node metastases	0.99 (0.82-1.20)	0.97	1.00 (0.82-1.22)	0.94	1.28 (1.11-1.46)	<0.001	1.02 (0.87-1.18)	0.79
Liver metastases	0.94 (0.78-1.14)	0.56	0.96 (0.78-1.17)	0.72	0.45 (0.39-0.52)*	<0.001	0.42 (0.36-0.49)*	<0.001
Peritoneal metastases	0.60 (0.43-0.85)*	0.004	0.63 (0.44-0.89)*	0.009	0.39 (0.31-0.50)*	<0.001	0.35 (0.27-0.46)*	<0.001
Lung metastases	1.28 (1.04-1.57)*	0.02	1.19 (0.97-1.47)	0.09	0.94 (0.81-1.09)	0.43	0.89 (0.76-1.06)	0.20
Bone metastases	0.93 (0.74-1.17)	0.57	0.94 (0.74-1.20)	0.64	0.92 (0.78-1.09)	0.36	0.84 (0.70-1.01)	0.08
Other sites	0.89 (0.70-1.14)	0.38	0.93 (0.72-1.20)	0.60	0.64 (0.53-0.77)*	<0.001	0.63 (0.52-0.77)*	<0.001
**Characteristics of the hospital of diagnosis**								
Center of expertise								
Yes	0.95 (0.77-1.16)	0.64	0.93 (0.75-1.15)	0.52	1.06 (0.91-1.22)	0.41	1.01 (0.85-1.19)	0.88
Hospital volume								
Q1 (≤68)	Ref		Ref		Ref		Ref	
Q2 (69-107)	1.53 (0.98-2.38)	0.06	1.52 (0.97-2.38)	0.06	0.67 (0.50-0.89)*	0.007	0.62 (0.45-0.85)*	0.003
Q3 (108-169)	1.00 (0.64-1.54)	0.99	0.99 (0.64-1.54)	0.99	0.72 (0.55-0.95)*	0.02	0.72 (0.53-0.98)*	0.04
Q4 (≥170)	1.39 (0.92-2.11)	0.11	1.43 (0.94-2.18)	0.10	0.65 (0.49-0.85)*	0.002	0.55 (0.41-0.75)*	<0.001

*<=10% meaning that patients' weight at diagnosis was 10% or less below their usual weight. >10% meaning that the patient's weight at diagnosis was more than 10% below their usual weight.

Table S2: Logistic regression analyses for the probability of receiving symptom-directed treatment in patients with metachronous metastatic esophageal cancer who received no systemic treatment.

	Esophageal cancer			
	Univariable		Multivariable	
	OR (95%CI)	P-value	OR (95%CI)	P-value
**Patient characteristics at diagnosis**				
Age	1.00 (1.00-1.01)	0.03	1.01 (1.00-1.01)	0.002
Sex				
Male	Ref		Ref	
Female	0.92 (0.80-1.07)	0.33	0.91 (0.78-1.08)	0.31
Weight loss				
<= 10%	Ref		Ref	
>10%	0.89 (0.74-1.07)	0.24	0.96 (0.74-1.17)	0.73
Unknown	0.66 (0.56-0.76)	<0.001	0.80 (0.68-0.95)	0.01
Comorbidities				
0	Ref		Ref	
1	1.13 (0.97-1.32)	0.09	1.08 (0.92-1.28)	0.31
≥2	1.07 (0.90-1.26)	0.40	1.01 (0.84-1.21)	0.91
Unknown	0.82 (0.58-1.16)	0.27	1.00 (0.68-1.46)	0.97
Performance status				
0-1	Ref		Ref	
≥2	0.53 (0.44-0.61)	<0.001	0.53 (0.44-0.63)	<0.001
Unknown	0.31 (0.27-0.37)	<0.001	0.32 (0.35-0.38)	<0.001
**Disease characteristics at diagnosis**				
Histology				
Adenocarcinoma	Ref		Ref	
Squamous cell carcinoma	1.76 (1.47-2.11)	<0.001	1.52 (1.25-1.85)	<0.001
Other	0.65 (0.47-0.90)	0.01	0.70 (0.49-0.99)	0.05
<u>Localization of metastases</u>				
Extraregional lymph node metastases	1.20 (1.05-1.37)	0.004	1.01 (0.88-1.17)	0.79
Liver metastases	0.48 (0.42-0.55)	<0.001	0.49 (0.45-0.56)	<0.001
Peritoneal metastases	0.42 (0.24-0.52)	<0.001	0.40 (0.32-0.50)	<0.001
Lung metastases	1.05 (0.91-1.22)	0.42	1.03 (0.88-1.20)	0.67
Bone metastases	1.44 (1.23-1.69)	<0.001	1.43 (1.20-1.69)	<0.001
Other sites	0.78 (0.66-0.92)	0.003	0.76 (0.64-0.91)	0.003
**Characteristics of the hospital of diagnosis**				
Center of expertise				
Yes	1.02 (0.88-1.17)	0.75	0.97 (0.83-1.14)	0.78
Hospital volume				
Q1 (≤68)	Ref		Ref	
Q2 (69-107)	0.88 (0.66-1.17)	0.38	0.87 (0.64-1.18)	0.34
Q3 (108-169)	0.80 (0.61-1.05)	0.11	0.80 (0.60-1.07)	0.14
Q4 (≥170)	0.78 (0.60-1.01)	0.07	0.69 (0.52-0.93)	0.01

Table S3: Characteristics of patients with advanced gastric and esophageal cancer diagnosed in 2015-2021 who received and who did not receive systemic treatment (matched cohort).

	**Esophageal cancer**			**Gastric cancer**		
	**Systemic therapy**	**No systemic therapy**		**Systemic therapy**	**No systemic therapy**	
	**N (%)**	**N (%)**	**P-value**	**N (%)**	**N (%)**	**P-value**
Number of patients	480	480		230	230	
**Patient characteristics at diagnosis**						
Age; mean (SD)	69 (63-74)	68 (61-75)	0.78	70 (64-76)	71 (62-77)	0.95
Hemoglobin (mmol/L); median (IQR)	8 (7-9)	8 (7-9)	0.22	8 (7-8)	7 (6-9)	0.08
LDH (U/L); median (IQR)	203 (173-251)	214 (180-285)	0.02	212 (174-299)	209 (177-304)	0.52
Weight loss (kg); median (IQR)	6 (4-10)	7 (3-10)	0.92	7 (3-10)	7 (3-10)	0.82
Sex						
Male	375 (78)	380 (79)	0.69	131 (57)	141 (61)	0.34
Female	105 (22)	100 (21)		99 (43)	89 (39)	
Comorbidities						
0	238 (50)	239 (50)	0.92	117 (51)	113 (49)	0.77
1	160 (33)	155 (32)		78 (34)	85 (37)	
≥2	82 (17)	86 (18)		35 (15)	32 (14)	
Performance status						
0-1	358 (75)	354 (74)	0.76	149 (65)	151 (66)	0.84
≥2	122 (25)	126 (26)		81 (35)	79 (34)	
**Disease characteristics at diagnosis**						
Disease type						
Unresectable advanced	15 (3)	11 (2)	0.69	10 (4)	10 (4)	0.79
Synchronous metastatic	446 (93)	448 (93)		211 (92)	208 (90)	
Metachronous metastatic	19 (4)	21 (4)		9 (4)	12 (5)	
Histology						
Adenocarcinoma	360 (75)	367 (76)	0.33	226 (98)	221 (96)	0.16
Squamous cell carcinoma	100 (21)	86 (18)		NA	NA	
Other	20 (4)	27 (6)		4 (2)	9 (4)	
Lauren classification						
Intestinal	237 (49)	248 (52)	0.96	67 (29)	76 (33)	0.50
Diffuse	93 (19)	91 (19)		145 (63)	133 (58)	
Mixed	9 (2)	9 (2)		7 (3)	5 (2)	
Indeterminate	21 (4)	19 (4)		7 (3)	7 (3)	
Not applicable (SCC)	120 (25)	113 (24)		4 (2)	9 (4)	
Distant metastatic sites						
0-1	292 (61)	287 (60)	0.72	163 (71)	150 (65)	0.42
2	110 (23)	120 (25)		50 (22)	59 (26)	
≥3	78 (16)	73 (15)		17 (7)	21 (9)	
Localization of metastases (not mutually exclusive)						
Extraregional lymph node metastases	248 (52)	243 (51)	0.74	72 (31)	78 (34)	0.55

Liver metastases	217 (45)	229 (48)	0.43	51 (22)	61 (27)	0.27
Peritoneal metastases	60 (13)	62 (13)	0.84	150 (65)	149 (65)	0.92
Lung metastases	122 (25)	111 (23)	0.41	16 (7)	20 (9)	0.48
Bone metastases	103 (22)	100 (21)	0.81	17 (7)	20 (9)	0.60
Other sites	88 (18)	97 (20)	0.46	16 (7)	19 (8)	0.59

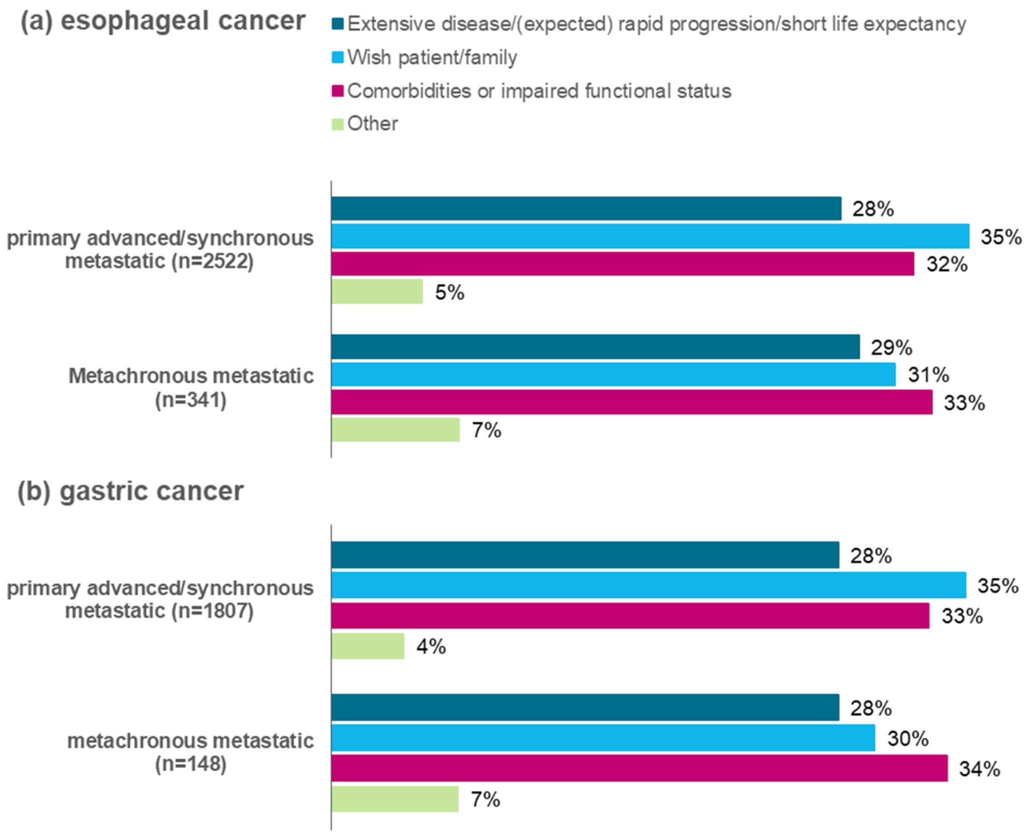

Figure S1: Main reason for forgoing treatment in patients with advanced esophageal (a) or gastric (b) cancer stratified by type of disease (primary advanced/synchronous metastatic or metachronous metastatic. Data are shown only for patients for whom the reason was recorded.

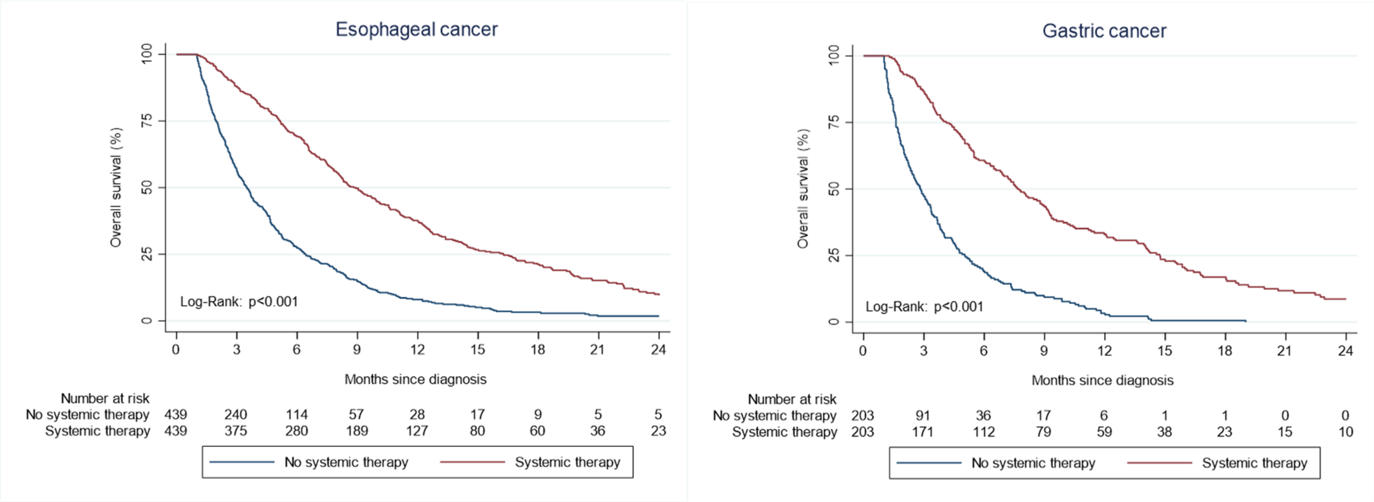

Figure S2: Kaplan Meijer curves of overall survival in patients with advanced esophagogastric cancer who received systemic treatment and who did not receive systemic treatment in a matched cohort with similar prognostic profiles at diagnosis, excluding patients who died within one month of diagnosis.
